# Urinary collagen degradation products as early markers of progressive renal fibrosis

**DOI:** 10.1186/s12967-017-1163-2

**Published:** 2017-03-20

**Authors:** Ryanne S. Hijmans, Daniel Guldager Kring Rasmussen, Saleh Yazdani, Gerjan Navis, Harry van Goor, Morten Asser Karsdal, Federica Genovese, Jacob van den Born

**Affiliations:** 1 Division of Nephrology, Department of Medicine, University Medical Center Groningen, Groningen, The Netherlands; 2 Division of Pathology, Department of Pathology and Medical Biology, University Medical Center Groningen, Groningen, The Netherlands; 3 Nordic Bioscience, Biomarkers & Research, Herlev, Denmark; 4 Institute of Molecular Medicine, Cardiovascular and Renal Research, Institute of Clinical Research, University of Southern Denmark, Odense, Denmark

**Keywords:** Fibrosis, Collagen, MMP, Biomarker, Chronic kidney disease, Kidney

## Abstract

**Background:**

Renal fibrogenesis is associated with increased ECM remodeling and release of collagen fragments in urine in progressive renal disease. We investigated the diagnostic value of urinary collagen degradation products in a proteinuria-driven fibrosis rat model with and without anti-fibrotic S1P-receptor modulator FTY720 treatment.

**Methods:**

Proteinuria was induced in male Wistar rats by Adriamycin (ADR) injection (n = 16). Healthy rats served as controls (n = 12). Six weeks post-injection, all underwent renal biopsy, and FTY720-treatment started in ADR-rats (n = 8) and controls (n = 6). Others remained untreated. Rats were sacrificed after 12 weeks. Collagen type I (C1M) and III (C3M) degradation fragments were measured in blood and urine using ELISA. Kidneys were stained for various inflammatory and fibrotic markers.

**Results:**

Six weeks post-injection proteinuria increased (versus controls, P < 0.001) and although no accumulation of interstitial renal collagen type III (iColl3) was observed at this time, urinary C3M (uC3M) and C1M (uC1M) were significantly increased (both P < 0.001). At 12 weeks, uC3M (P < 0.001) and uC1M (P < 0.01) further increased in ADR-rats versus controls, just as fibronectin, PDGF-β receptor, hyaluronan (all P < 0.01), iColl3, PAS, myofibroblasts, macrophages and T-cells (all P < 0.05). FTY720-treatment reduced accumulation of immune cells, α-SMA+ myofibroblasts and PAS-score, but not iColl3 and uC3M. Correlation analyses indicated that uC3M and uC1M reflected and predicted tubulointerstitial fibrogenesis.

**Conclusions:**

These data displayed urinary collagen breakdown products as sensitive early markers of interstitial fibrosis, preceding histological fibrotic changes, which might replace the invasive renal biopsy procedure to assess fibrosis. Anti-fibrotic FTY720 intervention reduced some fibrotic markers without affecting collagen type III metabolism.

## Background

Renal interstitial fibrosis (IF) is an important pathological feature of disease progression in chronic kidney disease (CKD) [1, 2]. Despite its importance as a marker of disease progression, early detection and quantitative analysis of IF remains a challenge, both in the clinical and in the experimental setting. The most widely used and accepted clinical markers to predict the progression of CKD are the estimated glomerular filtration rate (eGFR) and albuminuria [3–5]. By the time these functional changes are detectable, advanced pathological structural changes have already taken place. Therefore it would be beneficial to be able to detect renal tissue remodeling earlier [6].

Currently, the gold standard to assess renal IF in CKD is performing a renal biopsy followed by a semi-quantitative histological evaluation by a pathologist. This approach is however prone to sampling error and to intra- and inter-observer variability [7]. Many studies have tried to find non-invasive, more sensitive and IF specific alternatives. One of the most promising alternatives are urinary markers, which are consequent of the processes of extracellular matrix (ECM) production and degradation, thus reflecting fibrogenesis [6, 8].

Renal IF is characterized by an increased production and deposition of ECM, which eventually leads to a progressive loss of kidney function [9]. For tissue homeostasis, it is important to maintain a balance between synthesis and degradation of ECM proteins. However, this equilibrium is delicate and when disrupted, it can lead to IF [10].

In terms of synthesis, (myo)fibroblasts with an activated phenotype expressing smooth muscle actin (α-SMA) are considered to be the main source of the increased deposition of ECM [11–15]. Earlier studies showed that interstitial fibrosis is the result of an increase in important ECM components, namely collagen type I, collagen type III, fibronectin and proteoglycans [16–18].

Collagen type I and III are major interstitial collagens and their turnover is determined by, among other enzymes, matrix metalloproteinases (MMP), which are zinc-dependent enzymes and are synthesized in many tissues including the kidney [19–22]. During tissue remodeling, small protein fragments are released into the circulation, where these may be used as biomarkers. Two promising markers are generated by MMP-mediated collagen type I and III degradation (C1M and C3M, respectively) which have been shown to reflect renal IF [23–25].

In this study, our aim was to assess the diagnostic value for fibrosis of different collagen degradation products in the urine (uC1M and uC3M) and plasma (pC3M), as compared to (immuno)histological markers of fibrosis in a rat model of Adriamycin-induced nephropathy (i.e. a model of proteinuria-induced tubulointerstitial fibrosis). S1P receptor modulator FTY720 (FINGOLIMOD^®^) has been shown to have an inhibitory effect on fibrosis and on T cell mediated inflammation [26–29]. Therefore this intervention was used to distinguish the effects of anti-fibrotic treatment on different components of ECM remodeling pathways in the kidneys, as well as on collagen fragments in plasma and urine.

In our well-established rat model proteinuria precedes fibrosis and allows prognostic evaluation of fibrotic markers in relation to the deposition and prevention of fibrosis.

## Methods

### Animal experiment and treatments

The detailed experimental setting has been described previously [30]. In short, twenty-eight 3-month old male Wistar rats were randomly divided in two groups. In the first group (ADR-rats; N = 16) proteinuria was induced by injecting Adriamycin in the tail vein (2 mg/kg body weight). The rats in the second group received a saline injection in the tail vein and served as healthy controls (HC; N = 12). Six weeks after the injections, renal biopsies were taken to evaluate renal structural damage as described before [31]. After 3 days of recovery, treatment with S1P-receptor modulator FTY720 (Novartis, Basel, Switzerland, 1 mg/kg body weight per day in drinking water) was started in the ADR group (ADR-FTY; N = 8) and in the control group (C-FTY; N = 6). Eight ADR-rats and six control rats remained untreated.

At 12 weeks, blood pressure was measured under general anesthesia with the Cardiocap/5 (Datex-Ohmeda, Newark, USA). Animals were then sacrificed and organs were harvested after saline perfusion. Renal tissue obtained from the biopsy (6 weeks) and at sacrifice (12 weeks) was collected and partly preserved in formaldehyde 10% for paraffin embedment, and also snap-froze stored at −80 °C for cryosectionting and qRT-PCR analyses.

At the beginning of the study, at the time of the biopsy (6 weeks), and at the end of the experiment (12 weeks), bodyweight was measured, blood samples were collected and rats were placed in metabolic cages for 24 h urine collection and the measurement of food and water intake. Proteinuria was determined in urine samples by a turbidimetric assay (Roche Modular P, Mannheim,Germany). Urea and creatinine in plasma and urine were measured by an enzymatic UV assay (Roche Modular P).

The experiment was carried out under a protocol, which was approved by the Animal Care Committee of the University of Groningen (Licence Number 6318D).

### Immunohistochemistry

Immunohistochemical staining was performed on 4-μm-thick formalin-fixed paraffin sections after deparaffinization in xylene and rehydration in alcohol series, or on 4-μm-thick cryo sections with acetone fixation. For paraffin sections; antigen retrieval was done for 15 min in a microwave oven for Tris/EDTA buffer pH:9.0 and citrate buffer pH:6.0, or overnight at 80 °C in Tris/HCl buffer pH:8.0. For paraffin and cryo sections; endogenous peroxidase activity was blocked with 0.3 or 0.03% hydrogen peroxide, respectively. Endogenous biotin binding sites were blocked by an Avidin/Biotin blocking step in case of biotin-labeled first antibodies. Sections were incubated for 1 h or overnight at 4 °C with the following primary antibodies: mouse anti-human α-smooth muscle actin (SMA) (clone 1A4, Sigma-Aldrich, St Louis, USA), goat anti-collagen type III (cat. no. 1330-01, Southern Biotech, Birmingham, USA), rabbit anti-rat CD3 (clone A0452, Dako, Glostrup, Denmark), biotinylated hyaluronan binding protein (HABP, Seikagaku, Tokyo, Japan), mouse anti-rat CD68 (clone ED1, AbD Serotec, Oxford, UK), and rabbit mAb anti-platelet-derived growth factor (PDGF) Receptor β (28E1) (cat. No. 3169, Cell Signaling Technology, Danvers, USA). After this step, the sections were incubated with secondary and tertiary antibodies diluted in PBS/1% BSA and 1% normal rat serum. We used rabbit anti-mouse Ig horseradish peroxidase (HRP), goat anti-rabbit Ig HRP, goat anti-mouse Ig HRP, rabbit anti-goat Ig HRP, swine anti-rabbit HRP and anti-rabbit poly HRP (all from Dako, Glostrup, Denmark). As negative controls, the primary antibodies were replaced by PBS/1% BSA. The negative controls were found to be negative. Bound antibodies were visualized by aminoethylcarbazole (AEC) or by the peroxidase substrate 3,3-diaminobenzidine (DAB) (Sigma-Aldrich, St Louis, USA) and then counterstained with diluted hematoxylin. Biotinylated HABP was visualized using FITC-conjugated streptavidin (Invitrogen, Carlsbad, USA). DAPI (Vector laboratories, Burlingame, USA) was used to stain nuclei. The sections were scanned with a NanoZoomer HT digital scanner (Hamamatsu Photonics K.K., Shizuoka Pref., Japan). Fluorescence microscopy was performed using a Leica DMLB microscope (Leica Microsystems, Rijswijk, the Netherlands) equipped with a Leica DC300F camera and Leica Qwin 2.8 software. The quantification was done using Aperio ImageScope software (version 9.1.772.1570, Aperio Technologies Inc., Vista, CA, USA) and ImageJ 1.46r (Rasband, W.S., U.S. National Institutes of Health). ED1-positive macrophages and CD3-positive T cells were manually counted in 30 cortical interstitial fields per kidney. The expression of collagen type III, hyaluronan, PDGF-β receptor and myofibroblasts (α-SMA) was measured by using an automatic quantification method using ImageJ 1.41 (Rasband, W.S., U.S. National Institutes of Health) and expressed as a % of positively stained area.

### Renal morphology

Sections (4 μm) of formalin-fixed paraffin embedded kidneys were stained with Periodic Acid Schiff (PAS). Renal damage was semi-quantitatively scored on a scale ranging from 1 to 5. The scoring indicates which part of total renal cortical tissue was affected by tubulointerstitial fibrosis (PAS-positive broadening interstitial area in between the tubules): score 1: <1%; score 2: 1–5%; score 3: 6–10%; score 4: 11–20%; score 5: 21–50%.

### Use of published data by our group

The data on collagen III, alpha-SMA and PAS from Fig. 2 were published before [30], however are necessary for comparison with the new stainings and quantifications of the other markers of fibrosis, namely fibronectin, PDGF Receptor beta and hyaluronan (see Fig. 2). Since the same authors are involved in both the previous as well as in the writing of this manuscript, permission was granted to use the data.

### Plasma and urinary collagen degradation products

Supernatant from antibody producing hybridoma was collected and the monoclonal antibody was purified using HiTrap affinity columns (GE Healthcare Life Science, Little Chalfront, Buckinghamshire, UK) and labeled with HRP using Lightning-Link^™^ HRP Conjugation Kit (Innova Biosciences, Babraham, Cambridge, UK), according to the manufacturer’s instructions.

The competitive ELISA procedures have been described in detail before [23, 24]. In short, procedures were as follows: Streptavidin coated plates were incubated with 100 µl biotinylated-peptide for 30 min at 20 °C. Plates were washed five times in washing buffer (20 nM TRIS, 50 mM NaCl, pH 7.2). Sample/standard/control (20 µl) was added and followed immediately by addition of 100 µl HRP labeled monoclonal antibody and incubated for 1 h at 20 °C (pC3M), 3 h at 4 °C (uC1M), or 20 h at 4 °C (uC3M). After incubation, plates were washed five times in washing buffer. A volume of 100 µl 3,3′,5,5′ -Tetramethylbenzidine (TMB) was added and incubated for 15 min at 20 °C in the dark. To stop the enzyme reaction of TMB, 100 µl 0.1% sulphuric acid was added and the plate was analyzed in the ELISA reader at 450 nm with 650 nm as the reference (Molecular Devices, SpectraMax M, CA, USA). A calibration curve was plotted using a 4-parametric mathematical fit model. Each ELISA plate included kit controls to monitor inter-assay variation. All samples were measured within the range of the specific assay. All samples below the lower limit of quantification (LLOQ) were assigned the value of LLOQ. The urinary markers were normalized for urinary creatinine.

### Statistical analyses

Statistical analysis was performed using SPSS 20.0 (SPSS Inc., Chicago, IL, USA) or Statsdirect 3.0 (Cheshire, UK), and GraphPad Prism 5.0 (GraphPad Software Inc., La Jolla, CA, USA) was used to construct graphs and figures. Statistical differences between all groups at the same time-point were tested using the non-parametric unpaired Kruskall-Wallis test (with Dwass-Steel-Critchlow-Fligner post-test) and differences over time by using the non-parametric paired Friedmann test (with Iman and Davenport post-test). Correlations were tested using the non-parametric Spearmann Rank test on the Z-scores of the variables used. Correlations between collagen degradation markers at 12 weeks and histological markers at 6 weeks were tested using data of untreated ADR-rats only (N = 8). For correlations at between variables at the same time-point (12 weeks), we paired the FTY720-treated ADR-rats and the untreated ADR-rats (N = 16). Statistical differences of P < 0. 05 were considered significant.

## Results

### Adriamycin induced proteinuria and tubulointerstitial remodeling

The effects of ADR on proteinuria and tubulointerstitial remodeling have been described in detail before [30]. In short a summary of the relevant results for our study: 6 weeks after ADR injection proteinuria was increased eightfold up to 146 [77–230] mg/24 h (P < 0.001) compared to controls (18 [13–27] mg/24 h). At this early time-point, tubulointerstitial myofibroblasts started to appear, however, no interstitial collagen type III deposition was observed yet. Twelve weeks after ADR injection, proteinuria further increased up to 338 [176–535] mg/24 h, which was associated with an increase in tubulointerstitial fibrosis (PAS), increased collagen type III deposition, and accumulation of myofibroblasts, macrophages (MΦ) and T-cells (all P < 0.05). Treatment of ADR-rats from weeks 6 to 12 with FTY720 neither reduced proteinuria nor reduced renal collagen type III expression (both at protein and mRNA level). However, renal accumulation of myofibroblasts and T-cells were significantly lower after FTY720 treatment (both P < 0.05) and apparently MΦ (NS). FTY720 also significantly reduced tubulointerstitial fibrosis score (PAS; P < 0.05).

### Longitudinal analysis of plasma and urinary collagen degradation fragments

To assess the diagnostic and predictive value of non-invasive collagen degradation fragments, we time-dependently analysed the markers of ECM remodeling in healthy control rats and ADR-injected rats, both untreated and treated with S1P-receptor modulator FTY720.

**Fig. 1:**
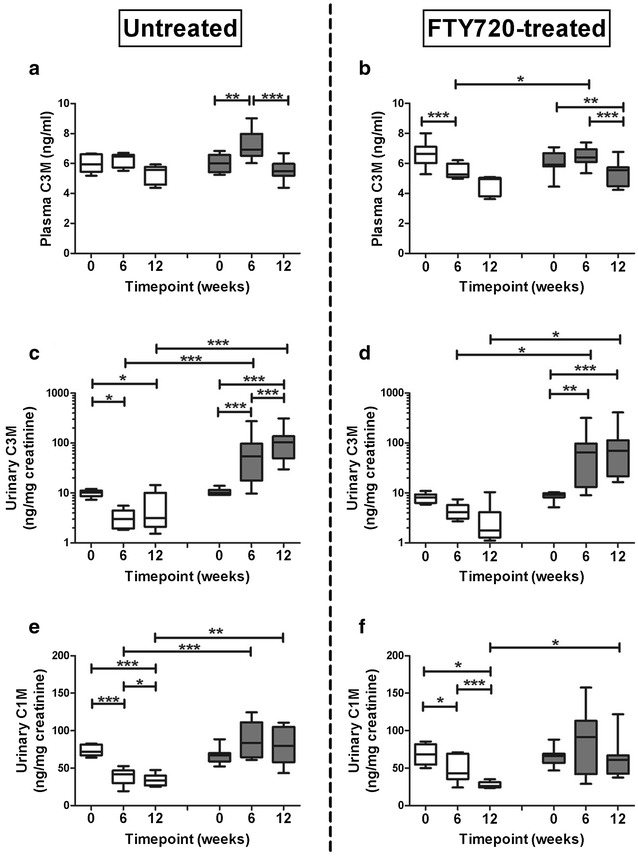
Effects of Adriamycin with or without FTY720 treatment on collagen degradation markers. Effects of Adriamycin are shown without (*left panels*) and with FTY720 treatment (*right panels*) on pC3M (**a**, **b**), uC3M (**c**, **d**), and uC1M (**e**, **f**). uC3M and uC1M concentrations were expressed per mg urinary creatinine. *Open boxes* represent controls, *dark grey boxes* ADR-rats. *P < 0.05, **P < 0.01, ***P < 0.001. Statistical differences between all groups at the same time-point were tested using the Kruskall-Wallis test (Dwass-Steel-Critchlow-Fligner post-test) and differences over time by using the Friedmann test (Iman and Davenport post-test)

The timecourse of untreated ADR-rats (Fig. 1. Left panels), showed a transient increase of pC3M 6 weeks after ADR injection (baseline vs week 6: P < 0.01), that returned to baseline values at 12 weeks (Fig. 1a; weeks 12 vs 6: P < 0.001). uC3M values showed an ongoing increase at weeks 6 and 12 in ADR-rats compared to control values (Fig. 1c; both P < 0.001). uC1M levels were significantly higher both at weeks 6 (P < 0.001) and 12 (P < 0.01) in ADR-rats compared to their healthy controls at the same time-points (Fig. 1e). These data showed increased excretion of uC1M and uC3M upon progression of proteinuria-induced tubulointerstitial fibrosis.

Treatment of healthy controls with FTY720 (Fig. 1. Right panels) showed a decreasing trend in pC3M over time, while FTY720-treated ADR-rats showed a significant decrease at 12 weeks compared to baseline (P < 0.01) and 6 weeks (Fig. 1b; P < 0.001). uC3M levels in the FTY720-treated ADR-rats (Fig. 1d) increased significantly over time at both 6 weeks (P < 0.01) and 12 weeks (P < 0.001) compared to baseline values. uC1M levels in healthy controls treated with FTY720 showed a significant decrease over time at 6 (P < 0.05) and 12 weeks (P < 0.001). At 12 weeks, FTY720-treated ADR-rats showed significantly higher uC1M values compared to their controls at 12 weeks (Fig. 1f; P < 0.05). Importantly, FTY720 treatment did not reduce excretion of both uC3M and uC1M in ADR-rats.

To dissect different fibrotic pathways, we also looked at specific fibrotic markers in the renal tissue and if these markers are affected by treatment with FTY720. At 6 weeks, we did not see any significant changes for collagen type III expression, α-SMA positive myofibroblasts and tubulointerstitial fibrosis (PAS) in the renal tissue, which has been published before by our group and is needed to interpret our most recent findings [30].

**Fig. 2:**
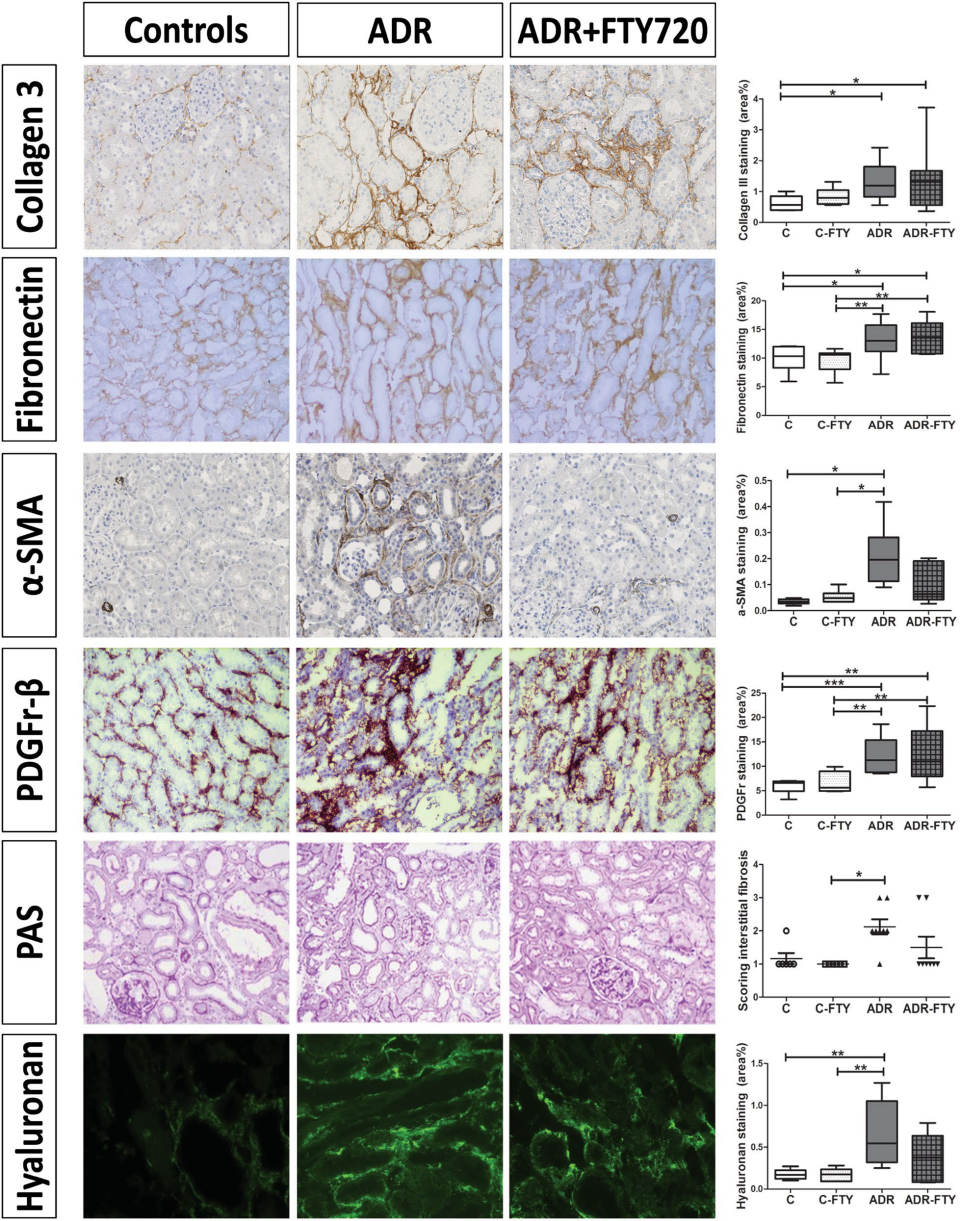
Effects of FTY720 treatment on tubule-interstitial remodeling at week 12. Expression of interstitial collagen type III (×400), fibronectin (×200), α-SMA positive myofibroblasts (×200), PDGF-β receptor (×200), interstitial fibrosis (PAS; ×200) and hyaluronan (×400) at week 12. Data of collagen type III, α-SMA and PAS have been published before [30]). Permission to show data was granted by the authors and publishers. *P < 0.05, **P < 0.01, ***P < 0.001. Statistical differences between groups were tested using the Kruskall-Wallis test (Dwass-Steel-Critchlow-Fligner post-test)

At 12 weeks, ADR-rats showed an increase in collagen type III expression (Fig. 2 first row; P < 0.05), α-SMA positive myofibroblasts (Fig. 2 third row; P < 0.05) and tubulointerstitial fibrosis by PAS staining (Fig. 2 fifth row; P < 0.05) compared to controls, while treatment with FTY720 showed a (non-significant) decrease of the expression of α-SMA positive myofibroblasts and tubulointerstitial fibrosis (PAS score), which can no longer be differentiated from FTY720-treated control rats. The expression of fibronectin showed the same trend as collagen type III expression increased significantly in ADR-treated rats compared to controls (Fig. 2 second row; P < 0.01). Treatment with FTY720 did not have an effect on the expression of fibronectin.

In order to investigate α-SMA positive myofibroblasts as a possible pro-fibrotic marker, we compared these findings to the expression of the PDGF-β receptor as an alternative phenotypic fibroblast marker. The expression of PDGF-β receptor was increased in ADR-rats compared to controls (Fig. 2 fourth row; P < 0.01), whereas α-SMA positive myofibroblasts influx decreased under treatment with FTY720, there was no significant difference in PDGF-β receptor expression between untreated ADR-rats and FTY720-treated ADR-rats.

To investigate if the apparent reduction in tubulointerstitial fibrosis (PAS) between untreated and FTY720-treated ADR-rats might be an effect of FTY720 on the accumulation of glycosaminoglycans instead of reflecting the collagen metabolism, a staining for hyaluronan was done. This staining showed the same non-significant decrease in the FTY720-treated ADR group as was shown by the PAS (Fig. 2 sixth row), corroborating that positive PAS staining represents glycosaminoglycans rather than collagens. Based on the quantification of various (immuno)histochemical markers we conclude that the FTY720 intervention tended to reduce accumulation of a-SMA positive myofibroblasts, hyaluronan and the fibrosis score based on PAS-staining. However, FTY720 treatment did not reduce the (myo)fibroblast marker PDGF-β receptor, interstitial collagen type III and fibronectin.

### uC3M forebodes the development of renal fibrosis

uC3M and uC1M at week 6 did not show any correlation with histological markers at the same time-point in all ADR-rats (not shown). However, uC3M at 6 weeks was associated with hyaluronan at 12 weeks and borderline with the PAS fibrosis score at 12 weeks in the untreated ADR-rats, but not with the other renal fibrosis markers (Table 1A). In addition, uC1M at 6 weeks associated borderline significance with the PAS-score at 12 weeks in untreated ADR-rats.

**Table 1:** Predictive value of uC3M and uC1M at 6 (A) weeks and correlation at 12 weeks (B) with histological fibrotic markers at week 12 in untreated ADR-rats and FTY720-treated ADR-rats

	uC3M/Cr 6 weeks		uC1M/Cr 6 weeks	
	R_s_	P value	R_s_	P value
A.				
ADR untreated rats				
Histological markers—12 weeks				
Collagen type III	0.381	0.352	0.262	0.531
Fibronectin	0.238	0.570	0.190	0.651
Myofibroblasts(α-SMA)	0.333	0.420	0.310	0.456
PDGF-β receptor	0.524	0.183	0.405	0.320
Tubulointerstitial fibrosis (PAS)	0.687	0.060	0.674	0.067
Hyaluronan	*0.810*	*0.015*	0.571	0.139

	uC3M/Cr 12 weeks		uC1M/Cr 12 weeks	
	R_s_	P value	R_s_	P value
B.				
ADR-rats (pooled)				
Histological markers—12 weeks				
Collagen type III	*0.529*	*0.035*	0.403	0.122
Fibronectin	0.074	0.787	0.194	0.471
Myofibroblasts(α-SMA)	*0.600*	*0.014*	*0.497*	*0.050*
PDGF-β receptor	*0.841*	<*0.0001*	*0.656*	*0.006*
Tubulointerstitial fibrosis (PAS)	*0.831*	*0.000*	*0.694*	*0.003*
Hyaluronan	*0.807*	*0.000*	*0.654*	*0.006*

Significant differences are highlighted in italics. Spearmann rank correlation was done on Z-values of all variables

At 12 weeks, after pooling of both the ADR-untreated as well as in FTY720-treated ADR-rats (N = 16), uC3M was strongly associated with renal collagen type III, myofibroblast density, PDGF-β receptor, tubulointerstial fibrosis (PAS) and hyaluronan. uC1M showed the same associations, except for collagen type III (Table 1B). These associations indicate that uC3M and uC1M represent and predict early development of renal fibrosis, at least in this experimental model of proteinuria-driven renal fibrosis.

## Discussion

The novel finding in this study is that uC3M is measurable prior to being histologically detectable, making it an early non-invasive predicting marker for renal fibrosis. Furthermore, collagen type I and III degradation (C1M and C3M) are late urinary markers reflecting the extent of established renal fibrosis. Dissection of different fibrotic pathways using S1P-modulator FTY720 showed that uC3M was more specific in reflecting renal fibrosis, in terms of collagen deposition, compared to the more commonly used PAS or α-SMA staining.

Proteinuria developed 6 weeks after Adriamycin injection in rats but collagen type III deposition was not observed at this time-point [30]. uC3M and uC1M were significantly elevated compared to the controls at 6 weeks. The uC3M results were profoundly elevated and therefore we measured plasma C3M as well and found a significant transient peak at 6 weeks compared to baseline and 12-week values. Karsdal et al. previously showed an age-related transient peak of pC3M at 8 weeks, which is around the same time-point where we found a transient peak of C3M as well (i.e. at 6 weeks) [32]. The data suggest that at 6 weeks, tissue remodeling had started and although no collagen deposition could be demonstrated at this early time-point, both urinary and plasma values of C1M and C3M were increased. This suggests active tissue remodeling, which most likely reflects a pre-fibrotic event. From weeks 6 to 12, interstitial fibrosis developed, uC1M remained elevated and uC3M increased even further. The reduction in the levels of uC3M and uC1M in the control untreated animals could be due to an age effect which has been described previously by Karsdal et al. [32]. Anti-fibrotic treatment with S1P modulator FTY720 (FINGOLIMOD^®^) yielded mixed results for the various fibrotic markers quantified in the kidneys. It appears that the kinetic response of the various fibrotic pathways varies with anti-fibrotic therapy. uC3M appears to be an early and sensitive marker of renal tissue remodeling.

In order to dissect different fibrotic pathways, we looked at the effect of anti-fibrotic treatment with FTY720 on different histological markers, which are frequently used (both in the clinic or for research purposes) to address renal fibrosis [7]. Previously published results on collagen type III expression, α-SMA positive myofibroblasts and tubulointerstitial expression (PAS), showed that treatment with FTY720 had different effects on these markers, even though all are known to reflect renal fibrosis [30]. The role of the extracellular matrix as a mere scaffold to uphold tissue integrity is being questioned, and it is becoming increasingly clear that it may be considered as a complex paracrine/endocrine entity [33]. Previous studies showed that structural proteins, such as collagens, do not only have organizational properties, but might also have other effects. For example, collagen type I, collagen type III and fibronectin are involved in signaling pathways during fibrosis and inflammation by activating hepatic stellate cells (reviewed in [33]). The dissection of the different components of ECM remodeling by anti-fibrotic and anti-inflammatory FTY720-treatment allows us to have a more specific look at the roles of the most important involved structural proteins.

To investigate these different outcomes and in order to define what kind of fibrotic pathway is reflected by increased uC3M, we furthermore performed staining for fibronectin (as a ECM marker which is able to bind to collagen), for the PDGF-β receptor (as an alternative fibroblast marker) and for hyaluronan (as a matrix glycosaminoglycan, GAG) [34–36]. Interestingly, fibronectin showed the same pattern as collagen type III. Findings were confirmed by previously published qPCR results, where treatment with FTY720 did not have a significant effect on collagen type I, collagen type III or TGF-β mRNA expression [30]. The expression of the PDGF-β receptor was unchanged after FTY720 treatment and clearly deviates from α-SMA positive myofibroblasts. There are different subpopulations of myofibroblasts and the finding that FTY720 lowered α-SMA positive myofibroblasts and did not have an effect on PDGF-β receptor-expressing fibroblasts suggests that there might be a shift in the population of fibroblasts or that the myofibroblasts lose α-SMA expression upon FTY720 treatment. PAS staining also showed a contrasting pattern compared to collagen type III expression after FTY720 treatment. PAS is used as a fibrotic marker, and specifically stains acid groups [37]. Therefore, we investigated if the decrease of PAS could be explained by a decreased glycosaminoglycan expression which are ECM compounds containing acid carboxyl groups. Staining was done for hyaluronan, which showed the same decrease after FTY720 treatment as PAS, confirming our hypothesis that the decrease of PAS staining after FTY720 treatment reflects a direct effect of FTY720 on the level of proteoglycans/GAGs.

The data suggest that treatment with FTY720 influences the differentiation of myofibroblasts by loss of α-SMA expression, without affecting the expression of the PDGF-β receptor. This showed no effect on the collagen and fibronectin metabolism. It however does influence GAGs as hyaluronan expression decreases and this consequently reduces the PAS fibrosis-score.

We were interested in the diagnostic and predictive value of uC3M and uC1M. At 6 weeks uC3M in the untreated ADR-rats showed a positive correlation with hyaluronan at 6 and at 12 weeks. At 6 weeks uC1M and uC3M were borderline significantly correlated with the PAS fibrosis score at 12 weeks. At 12 weeks uC1M and uC3M were associated with most of the fibrotic kidney markers at 12 weeks. These data indicated that early uC3M, and to a lesser extent uC1M, precede and reflect renal fibrosis.

The strength of this study is the well-established time course in which we were able to follow changes in both histological renal remodeling responses, as well as in urinary/plasma degradation products at different time-points. This allowed us to show that uC3M is elevated at an earlier time-point compared to histological collagen type III expression [6, 25]. Another strength is the dissection of the different fibrotic pathways by using FTY720 treatment, showing a deviation in collagen and hyaluronan tissue responses. We were thus able to show that uC3M reflects the level and predicts future deposition of type III collagen. Our data showed that uC3M is an early fibrotic marker for collagen type III deposition, but not representative for other ECM components.

During physiological conditions remodeling of the ECM is very low but increases during injury. Increased degradation is promoted by a rise in protease activity such as by MMPs [38]. Protease-generated fragments of a protein have been suggested to be more specific for pathological ECM turnover during fibrosis than total protein content since it is dependent on the local protease activity [39]. The C1M and C3M assays are highly specific which allows us to assess collagen degradation by MMPs, a process believed to be associated with the pathological turnover of the ECM during fibrosis [40]. To our knowledge, no comparable assay is currently available or published. Commercial assays for measurements of collagens in serum or urine are not directed against specific epitopes, but rather utilize polyclonal antibodies.

When we compare our results to what was found in humans, we found that uC3M decreases in humans with increasing disease severity, while it increases in rats [41]. The proces of fibrosis exist of both fibrogenesis and collagen degradation. In the early disease stages (as been shown in our animal model), fibrogenesis and collagen degradation balance eachother out and only when the disease progresses, fibrogenesis become more apperent compared to degradation and fibrotic laesions start to appear [42]. The fact that during disease progression the balance tips towards fibrogenesis can be explained by the fact that collagens are known to crosslink [43–45] and can become inaccessible for cleavage of collagens by MMPs [20, 38, 46]. Next, Di Donato et al. investigated the renal expression of lysyl oxidase and its effects on collagen cross-linking at various stages of chronic Adriamycin nephropathy in rats [47]. They showed that mRNA levels of lysyl oxidase increase up to 3 times in the ADR-rats compared to controls between 8 and 12 weeks, and the levels reduced to normal levels at 16 weeks. They demonstrated that an increased expression of lysyl oxidase in the kidney precedes the development of diffuse fibrotic lesions and that, at this stage, collagenic structures contain highly cross-linked components, the final product of lysyl oxidase activity. In our study, we only measured levels of collagen degradation markers between 6 and 12 weeks after ADR-injection, the period when lysyl oxidase levels are increasing, but collagen crosslinking has just begon and is not at his final stage. Lysyl oxidase has also been shown to be involved in fibrosis in humans [48, 49]. In patients with CKD the disease is present for longer then 6–12 weeks and collagen crosslinking is already occurring in abundance, making it hard for MMPs to degradate the collagens. This is reflected by the decreasing urinary C3M levels during disease progression [41]. In this study we are looking for a possible early marker for renal fibrosis, which could be the collagen degradation fragments in the urine, since they can already be found before the balance between renal fibrogenesis and collagen degradation is lost.

Possible limitations of this study are the previously used data, the pre-dominant interest in C3M in comparison to C1M, and the disease model. We have limited the re-use of our previously published data to the IHC data on collagen type III, PAS and α-SMA. However, we need this data to understand the new findings better. In our previous study [30] we first found these contrasting findings on collagen type III metabolism and the effects of anti-fibrotic FTY720 treatment on PAS and myofibroblasts. In this study, where we investigated if urinary collagen degradation fragments could be a good alternative for these invasive histological diagnostic markers for renal fibrosis, we found this very interesting novel finding that these urinary collagen degradation markers can be shown in the urine prior to any visible histological changes. While the need for good diagnostic, prognostic and next to this clinical applicable biomarkers is growing, we tried to investigate the different fibrotic pathways to further detail in order to get a better understanding of the specificity of the collagen degradation markers and the prognostic properties.

We found the most profound differences in the urinary C3M levels and the urinary C1M levels showed the same trends as urinary C3M. We choose to add the C1M data on urine to underline that the possibility of collagen degradation markers in the urine during renal fibrosis is not limited to collagen type III degradation fragments, but can also be found for collagen type I degradation fragments. At least in this study.

Next to this, we realize that the Adriamycin nephropathy model is driven by proteinuria. We therefore can not be absolutely certain whether the increased levels of uC1M and uC3M are due to increased glomerular filtration or if it is an effect of an increased turnover of renal type I and III collagen. However, in a previously published study collagen degradation fragments C1M and C3M were measured in three different rat models for nephropathy, namely renal mass reduction (5/6 nephrectomy), progressive glomerulonephritis (chronic anti-Thy1.1 nephritis) and adenine crystal-induced nephropathy. All three treatments caused significant renal fibrosis on a histological level and showed a significant increase of both uC3M and uC1M. Interestingly, while the adenine model caused the most profound increasing effect on the levels of uC3M and uC1M compared to the other models, it was not accompanied by proteinuria [25]. This underlines our hypothesis that the urinary collagen degradation fragments primarily reflect renal fibrogenesis and makes it unlikely that the increase in uC3M is mainly attributable to the proteinuria.

## Conclusions

In conclusion we described the greater diagnostic and predictive value of urinary collagen degradation markers C3M and C1M compared to the more conservative histological markers in a rat model of Adriamycin-induced nephropathy. We investigated the specificity of uC3M as a marker for renal fibrosis, by dissecting the collagen fibrotic pathway from other fibrotic pathways using anti-fibrotic S1P-receptor modulator FTY720. Although further research is needed to investigate the predictive value of the urinary collagen degradation markers, we propose that the diagnostic and predictive use of urinary collagen degradation markers to assess renal fibrosis can be used in early stages during disease progression of different etiologies. It might be used as a better, safer and more patient-friendly alternative than the renal biopsy, which is an invasive way of assessing histological markers of fibrosis.

## Abbreviations

ECM: extracellular matrix
ADR: adriamycin
iColl3: interstitial renal collagen type III
C1M: collagen type I degradation fragments
C3M: collagen type III degradation fragments
MMP: matrix metalloproteinases
α-SMA: anti-human α-smooth muscle actin
PDGF-β receptor: anti-platelet-derived growth factor receptor β
PAS: Periodic Acid Schiff
HABP: hyaluronan binding protein
